# Introduction to the study of chromosomal and reproductive patterns in Paraneoptera

**DOI:** 10.3897/compcytogen.v15.i3.69718

**Published:** 2021-07-19

**Authors:** Ilya A. Gavrilov-Zimin, Snejana M. Grozeva, Dmitrii A. Gapon, Andrei S. Kurochkin, Katia G. Trencheva, Valentina G. Kuznetsova

**Affiliations:** 1 Zoological Institute, Russian Academy of Sciences, Universitetskaya emb. 1, St. Petersburg, 199034, Russia; 2 Institute of Biodiversity and Ecosystem Research, Bulgarian Academy of Sciences, Blvd Tsar Osvoboditel 1, Sofia 1000, Bulgaria; 3 Samara National Research University, Moskovskoe Shosse, 34, Samara 443086, Russia; 4 University of Forestry, Blvd Kliment Ochridski 10, Sofia 1756, Bulgaria

**Keywords:** Aphids, cicadas, lice, moss-bugs, psocids, psyllids, scale insects, thrips, true bugs, whiteflies, zorapters

## Abstract

This paper opens the themed issue (a monograph) “Aberrant cytogenetic and reproductive patterns in the evolution of Paraneoptera”, prepared by a Russian-Bulgarian research team on the basis of long-term collaborative studies. In this first part of the issue, we provide the basic introductory information, describe the material involved and the methods applied, and give terminology and nomenclature of used taxonomic names.

## Introduction

The predominant reproductive strategy in eukaryotic organisms is bisexual reproduction which involves the formation and fusion of gametes, namely, sperm from the testes and eggs from the ovaries. This is also true for all major insect groups, in which, however, bisexuality is often combined with numerous aberrant modes of reproduction (White 1973; Ivanova-Kazas 1995; Simon et al. 2003; De Meeûs et al. 2007; Vershinina and Kuznetsova 2016; Leather and Hardie 2017; Gokhman and Kuznetsova 2018). These latter can characterize high-rank taxa or be found in separate genera and species within a group that mainly reproduces bisexually. The large insect supercohort Paraneoptera provides a unique opportunity to study almost the entire spectrum of aberrant reproductive strategies as well as genetic and chromosomal systems known in insects in general, such as ovoviviparity and viviparity, neoteny and paedogenesis, larval meiosis, achiasmate and inverted meiosis, parthenogenesis and polyploidization, dizygotic embryonal development, very peculiar types of mating, a huge variety of sex determination mechanisms, etc. It is important to point out that some reproductive patterns are often highly variable between or even within insect species.

**Figure 1.**
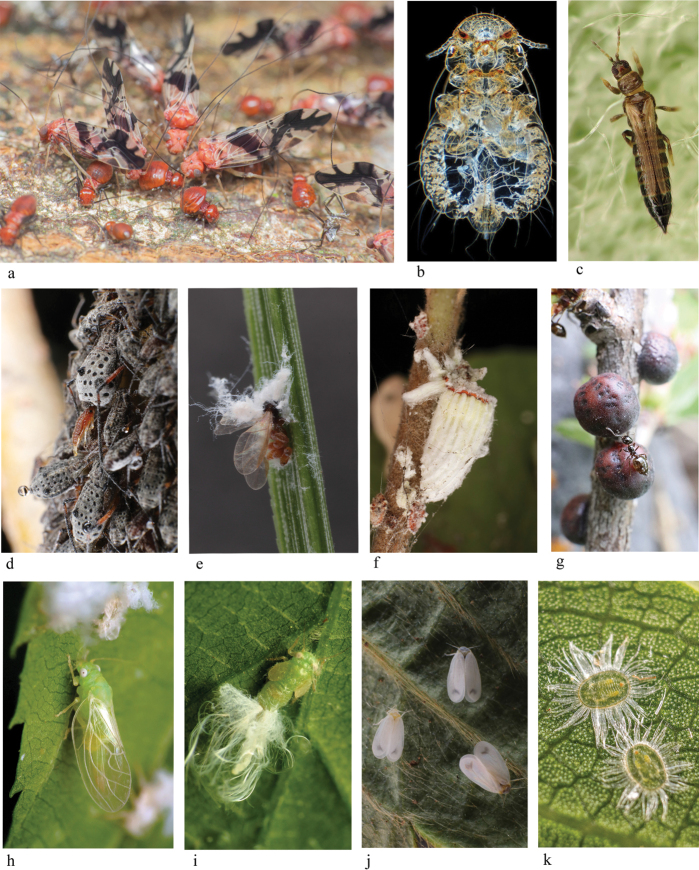
**a–k** Species from main taxonomic groups of Paraneoptera insects **a** imago and larvae of *Clematoscenea* sp. (Copeognatha), Singapore, photo and “Creative Commons” license of “Budak” (see Acknowledgements) **b** poultry fluff louse *Goniocotes gallinae* (de Geer, 1778) (Parasita), photo and “Creative Commons” license of “Da Re” (see Acknowledgements) **c** imago of *Taeniotrips inconsequens* (Uzel, 1895), Poland, photo and “Creative Commons” license of “Riszard” (see Acknowledgements) **d** colony of viviparous parthenogenetic females of *Tuberolachus silignus* (Gmelin, 1790) (Aphidinea), Samara Prov. of Russia, photo of A.S. Kurochkin **e** died female of *Adelges* sp. (Aphidinea) with developing eggs, Samara Prov. of Russia, photo of A.S. Kurochkin **f** adult female with wax ovisac and larvae of *Icerya purchasi* Maskell, 1879, Turkey, photo of A.S. Kurochkin **g** females of *Rhodococcus* sp. (Coccinea), attended by ant, Kazakhstan, photo of A.S. Kurochkin **h** imago and **i** larva of *Psylla carpinicola* Crawford, 1914 (Psyllinea), USA, photos and “Creative Commons” license of Katja Schulz (see Acknowledgements) **j** females and male of *Aleyrodes lonicerae* Walker, 1852 (Aleyrodinea), Samara Prov. of Russia, photo of A.S. Kurochkin **k** larvae of *Trialeurodes lauri* (Signoret, 1862), Turkey, photo of A.S. Kurochkin.

**Figure 2.**
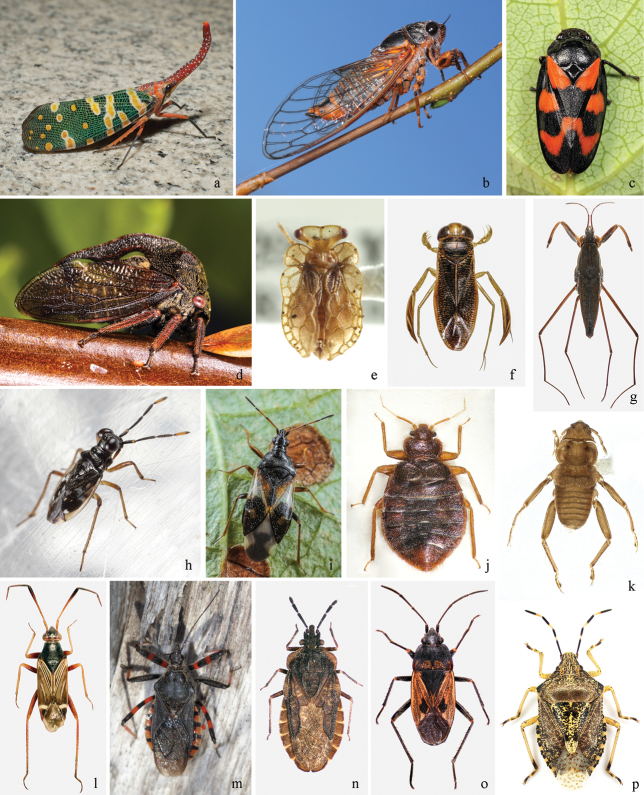
**a–p** Species from main taxonomic groups of Paraneoptera insects (continuation) **a***Pyrops candelaria* (Linnaeus, 1758) (Cicadinea, Fulgoridae), photo and “Creative Commons” license of “Sterling Sheehy” (see Acknowledgements) **b***Cicadetta montana* (Scopoli, 1772) (Cicadinea, Cicadidae), photo of E.Yu. Kirtsideli, PhD **c***Cercopis vulnerata* Rossi, 1807 (Cicadinea, Cercopidae), photo of E.Yu. Kirtsideli, PhD **d***Centrotus cornutus* (Linnaeus, 1758) (Cicadinea, Membracidae), photo of E.Yu. Kirtsideli, PhD **e***Pantinia darwini* China 1962 (Coleorrhyncha, Peloridiidae), photo and “Creative Commons” license of “Sterling Sheehy” (see Acknowledgements) **f***Glaenocorisa propinqua* (Fieber, 1860) (Heteroptera, Corixidae), photo of D.A. Gapon **g***Gerris sphagnetorum* Gaunitz, 1947 (Heteroptera, Gerridae) **h***Chartoscirta elegantula* (Fallén, 1807) (Heteroptera, Saldidae), photo of E.Yu. Kirtsideli, PhD **i***Anthocoris nemorum* (Linnaeus, 1761) (Heteroptera, Anthocoridae), photo of E.Yu. Kirtsideli, PhD **j***Cimex hemipterus* (Fabricius, 1803) (Heteroptera, Cimicidae), photo of D.A. Gapon **k***Hesperoctenes fumarius* (Westwood, 1874) (Heteroptera, Polyctenidae), photo and “Creative Commons” license of “CBG Photography Group” (see Acknowledgements) **l***Cremnocephalus albolineatus* Reuter, 1875 (Heteroptera, Miridae), photo of D.A. Gapon **m***Rhynocoris annulatus* (Linnaeus, 1758) (Heteroptera, Reduviidae), photo of E.Yu. Kirtsideli, PhD **n***Aradus laeviusculus* Reuter, 1875 (Heteroptera, Aradidae), photo of D.A. Gapon **o***Rhyparochromus phoeniceus* (Rossi, 1794) (Heteroptera, Lygaeidae), photo of D.A. Gapon **p***Rhaphigaster nebulosa* (Poda, 1761) (Heteroptera, Pentatomidae), photo of D.A. Gapon.

In this monograph, we attempt to summarize results of our own long-term investigations in the field and available literature data in order to give an overall picture of distribution of different reproductive characteristics within and among higher taxa of Paraneoptera. The supercohort Paraneoptera comprises about 130,000 recent species in the world fauna and is traditionally subdivided into the orders Zoraptera, Copeognatha (=Psocoptera), Parasita (=Phthiraptera), Thysanoptera, and the superorder Arthroidignatha (=Hemiptera sensu stricto) with two large orders, Heteroptera (true bugs) with about 45,000 species and Homoptera with about 66,000 species (Poisson and Pesson 1951; Henry 2017; Gavrilov-Zimin 2018; Kluge 2020) (Figs 1, 2). The last group is the most taxonomically diverse and combines five recent suborders, Aphidinea (about 5,000 species), Coccinea (8,000 species), Psyllinea (3,500 species), Aleyrodinea (1,500 species) and Cicadinea (47,000 species), which are quite divergent from each other morphologically, anatomically, cytogenetically, etc. Heteroptera compete with them in diversity and even surpass them in some aspects. This taxon is usually considered in the rank from order to suborder and, together with Coleorrhyncha (about 30 species), is often included in the higher taxon Heteropteroidea. Heteroptera are divided into seven infraorders, Enicocephalomorpha, Dipsocoromorpha, Nepomorpha, Gerromorpha, Leptopodomorpha, Cimicomorpha, and Pentatomomorpha; they include aquatic, semi-aquatic, surface-dwelling, terrestrial, carnivorous, blood-sucking, herbivorous, and parasitic representatives. The current level of knowledge of the cytogenetics and reproductive biology varies significantly between different groups of Paraneoptera. For example, among Aphidinea, Coccinea, Psyllinea, Cicadinea, and Heteroptera, several thousand species from all large families have been studied in this respect. On the other hand, among Zoraptera, Copeognatha, Parasita, Thysanoptera, and Aleyrodinea only occasional species from a small number of families have been analyzed, and both cytogenetic and reproductive characteristics of these groups are currently poorly known. The reason for this is largely due to difficulties in collecting these insects which are very small and lead a hidden life style.

We hope that our review issue will be useful for specialists in entomology, cytogenetics and evolutionary biology, as well as for those in the field of plant protection, veterinary and medicine. Many scale insects and aphids as well as some thrips, psyllids and true bugs are important pests of agricultural and ornamental plants and carriers of pathogenic viruses, whereas many lice and some true bugs are ectoparasites of invertebrate and vertebrate animals, including humans. Comparative knowledge of reproductive modes generated from studies across different phylogenetic lineages of Paraneoptera is essential for a better understanding of reproductive processes and underlying cytogenetic mechanisms in Insecta as a whole.

## Material and methods

The issue is based on the material collected mainly by authors and sometimes by their colleagues in different regions of the world including Western and Eastern Europe, Canary Islands, Morocco, Central Russia, Russian Far East, Crimea, Caucasus, Turkey, Myanmar, Thailand, Laos, Malacca peninsula, Sumatra, Java, Borneo, Sulawesi, New Guinea, Bali, and Flores Islands, Trinidad and Tobago, New Zealand, Tasmania, Vietnam and some others. The collections available at the Zoological Institute of the Russian Academy of Sciences (**ZIN RAS**, St. Petersburg) and the Institute of Biodiversity and Ecosystem Research of the Bulgarian Academy of Sciences (**IBER BAS**, Sofia) were also used.

Taxonomic identification of insects was mainly based on traditional morphological characters, with extensive use of the structural characters of the internal reproductive system and karyotype. In most cases, the identification was carried out using either intact insects, which are mounted on pins or mounting boards, or stored in ethanol/aceto-ethanol. To study male terminalia, the pygophore (genital segment) is detached from the abdomen and boiled for several minutes in 15–20% KOH solution. Parameres and aedeagus, removed from the pygophore using finest forceps and a dissecting needle, are examined at wet preparations. To study the structure of the endosoma (the internal membranous sac of the aedeagus), the method of its hydraulic inflation by means of glass microcapillaries is used, followed by drying the endosoma in a stream of hot air in a completely inflated state (Gapon 2001). The structure of the internal ectodermal parts of the female reproductive system is studied after boiling the abdomen in alkali and mechanical removal of soft tissues. Membranous structures on wet preparations are stained with methylene blue.

In the case of aphids, scale insects, whiteflies and thrips, permanent microscopic slides are prepared from insects and mounted in Canada balsam. Material processing included a different set of steps (procedures) depending on the object or on the specific purpose of the study. For example, in Thysanoptera, Aphidinea and Coccinea, studies were carried out on (ovo)viviparous females, eggs and larval instars of both sexes, respectively, whereas in Copeognatha, Parasita, Psyllinea, Cicadinea, Coleorrhyncha, and Heteroptera they were carried out mainly on males, although in some cases, females were also involved (when reproduction is parthenogenetic or when it is necessary to identify the chromosomal mechanism of sex determination in a particular species).

Preparation of permanent microscopic slides from aphids and coccids includes the following main steps (described in Gavrilov-Zimin 2018).

### Fixation

Insects, cleaned of plant tissues and/or soil particles, were fixed in 96% ethanol or (more often) in a mixture consisting of 1 part of glacial acetic acid and 3 parts of 96% ethanol (a Carnoy’s fixative). In our experience, the latter fixation is preferable due to the subsequent use of acid stains, for example, staining with acid fuchsin + pink lignin dissolved in Essig’s aphid fluid (see below). The use of acetoethanol also prevents superfluous dehydration of the fixed material. The volume of fixative must significantly (20 and more times) exceed the volume of the material. Fixed material is preserved in a dark place and, if possible, in a refrigerator. The minimum fixation time is 2–3 hours.

### Preliminary anatomizing

The insects were taken out of the fixative, put on the object glass in a drop of ethanol or distilled water and cut along the lateral body margin using a small blade.

### Clarification

The insects were placed in 8–15% water solution of sodium hydroxide (NaOH) or potassium hydroxide (KOH) and heated in a water bath or on any hot plate (about 60 °C) until the cuticle becomes translucent. The time of heating was selected experimentally for each object. With weakly sclerotized females (most mealybugs), 10–20 minutes of heating is usually enough. Heavily sclerotized and pigmented specimens demand 1–1.5 hours of heating. On the other hand, delicate soft females of Xylococcinae (Margarodidae s.l.) can be simply clarified in cold KOH or NaOH (about 20 °C) during several hours.

### Secondary anatomizing

Specimens were placed in a small amount of hot potash, and all internal organs were removed by light pressure on the cuticle using thin hooks. This is usually needed to change potash (hot or cold) several times until all the body internal content is removed. The specimens were then transferred to water for complete removing the potash.

### Staining

The most common method was originally developed for aphids (Essig 1948). The stain mixture consisting of 5 ml of acid fuchsin (4% water solution) and 10 ml of pink lignin (2% water solution) are dissolved in 100 ml of Essig’s fluid. The so-called “Essig’s fluid” can be prepared as follows: 20 parts of lactic acid (80–90% solution), 2 parts of phenol solution (1 gram phenol in 15 ml of distilled water), 4 parts of glacial acetic acid and 1 part of distilled water. Both mixtures are preserved separately in the refrigerator until use. Just before staining the material, several ml of Essig’s fluid should be poured in a small tube by adding 3–4 drops of stain mixture. Material can be stained directly in this tube for 20–30 min at 60 °C or for several hours at room temperature. Weakly sclerotized or poorly fixed specimens need longer staining. When successful staining of the object is reached, its sclerotized parts, such as antennae, legs, different setae and wax glands become well visible through the translucent background of the cuticle.

An older, but simpler and cheaper method of staining is based on the use of only fuchsin diluted in distilled water or in 96% ethanol until saturation. You can also take 1 g of basic fuchsin in 100 ml of 96% ethanol or 0.5 g of acid fuchsin in 25 ml of 10% water solution of 30% HCl and 300 ml of distilled water. The stain mixture by Dr. Jean-François Germain (Montpellier, France) consisting of acid fuchsin diluted until saturation in the mixture of distilled water, lactic acid (80–90% solution) and glycerol (1:1:1) also gives excellent results (Gavrilov-Zimin 2018).

After staining of any type, the material should be washed several times in 96% ethanol until the excess stain is removed.

### Oil impregnation

Canada balsam, which is usually used for the preparation of permanent slides, does not mix with water or ethanol. The specimens need, therefore, to be impregnated with an intermediate fluid, which can mix with both. This can be either a clove or bergamot oils, but other plant oils can also be tested if needed. The specimens should be placed in oil for 20–30 min while can be preserved in it for a longer time. If the acceptable oils are absent, it is possible (but undesirable!) to use xylene, toluene or something similar as an intermediate fluid. It is well known that a small amount of 96% ethanol can be mixed with a large amount of xylene or toluene. Therefore, the specimens can be get out of ethanol, air dried for several seconds and placed then in xylene or toluene for 20–30 min.

### Mounting

Following the oil or xylene/toluene impregnation, the specimens should be placed on a clean slide and excess oil must be removed with filter paper. Dorsal and ventral sides of the specimens, which were previously cut along the entire body margin, should be placed in the same plane. Then, a small drop of Canada balsam is dripping on the specimen(s) and covered with a cover slip. The slides are now ready for study, but care must be taken during several weeks until the slide is completely dried. Either thermostat or a drying box can be used to speed up drying. Dried slides can be stored in a dust-free place for an unlimited time at temperatures not higher than 35–40 °C.

Microscopic preparations were also prepared to study the reproductive biology, genetic systems and karyotypes of scale insects and aphids. With adult insects, both laid eggs and larval instars were fixed in acetoethanol (1:3) for at least 24 hours. The specimens were then dissected under a stereomicroscope and anatomized in a drop of 45% acetic acid. The simplest method for preparation of chromosome slides is based on staining with acetoorcein or acetocarmin or (better) with lactic acid solutions of these stains. This method gives acceptable results even for not well fixed material, being usually used for temporary slides only. For example, young embryos or gonads are stained by squashing in a drop of lactoacetorcein (50 ml 85% lactic acid : 2 g orcein : 50 ml glacial acetic acid). The cover slip can be fringed with rubber glue/cement, the slide will then be acceptable for study during a long time, especially if stored in the refrigerator. More complicated methods are based on staining with hematoxylin (see, for example, Dikshith 1964) or with Shiff’s reagent, the so-called Feulgen-Giemsa method described in Grozeva and Nokkala (1996). This method is widely used in cytogenetic studies of many insects including paraneopterans (see below); however, our experience with this method on scale insects and, in particular, on mealybugs, was negative. The main problem we encountered was an unpredictable influence of Shiff’s reagent on different species or even on different developmental stages of the same organ, which was previously noted also by other researches (see Romeis 1953).

The recent studies of chromosomes and internal reproductive organs of Paraneoptera insects other than Aphidinea and Coccinea, i.e. Zoraptera, Copeognatha, Parasita, Coleorrhyncha, Heteroptera, Cicadinea, and Psyllinea, were carried out using a different and wider range of methods and procedures.

### Sampling, fixation and storage of material

Male and female specimens, both newly emerged and older larvae, collected in the field, were fixed immediately in a freshly prepared Carnoy’s fixative (3:1) and refrigerated then in the laboratory at 4 °C until needed. If it was possible, some insects were brought to the laboratory alive. These were given a short hypotonic treatment with 1% tri-sodium citrate solution (Na_3_C_6_H_5_O_7_) for 5 min before the specimens were fixed in a fresh Carnoy’s.

### Study of the anatomy of testes in males and ovaries in females

The study was carried out on both live and fixed insects. In both male and female specimens, the abdomen was separated from the body and opened on a microscope slide in a drop of 45% acetic acid. The testes and ovaries were dissected out and analyzed under a stereomicroscope. In our anatomical research, we confined ourselves mainly to studying the number of testicular follicles and ovarioles, their shape and position on the sperm duct and oviduct, respectively. In separate cases, we studied the male internal reproductive system in general, with reference to the structure of the testes, presence/absence and shape of seminal vesicle(s), accessory glands and some additional associated structures. In psyllids and zorapters, we also analyzed the arrangement of spermatocytes within the follicle and the sequential stages of sperm formation. For this purpose, follicles were put on the slide in a drop of 45% acetic acid; coverslip was put on the drop and was allowed to settle without squashing. When all streaming was ceased, the slide was squashed gently allowing the spermatocytes to remain intact and retain their original location within the follicle.

### Study of chromosomes and meiosis

#### Slide preparation

Chromosome preparations from the male specimens (fixed in a Carnoy’s fixative) were made and stained as follows: testes were removed from the abdomen in 45% acetic acid. In some cases (when specimens were not fixed), they were removed in 1% tri-sodium citrate solution (Na_3_C_6_H_5_O_7_) for 5 min, fixed in a fresh Carnoy’s fixative and transferred then into a drop of 45% acetic acid on a slide. Testes were counted and cut into pieces (if large), mature sperms were largely removed, and squash preparations were made. The preparations were first examined by phase contrast to assess their quality and the presence of chromosomal divisions. After freezing off the coverslips in dry ice (a dry-ice technique by Conger and Fairchilld 1953), slides were dehydrated in fresh fixative solution for 30 min and air-dried. Chromosome preparations from female specimens were made and stained as follows: mature eggs were extracted from the abdomen and placed individually on slides in a drop of 45% acetic acid. After the chorion was removed and yolk became transparent, the eggs were squashed, and slides were made permanent by a dry-ice technique. In some cases (mainly in psyllid research), part of the material (both males and females) was both fixed and stored in 96% alcohol. In the laboratory, each of those specimens was dissected; the abdomen was immersed in the Carnoy’s fixative while the head and thorax part was stored in alcohol for subsequent sequencing. This allowed both chromosomal and haplotype (DNA barcoding) analyses of the same individual, which was very important for the purposes of accurate taxonomic identification of individuals (Nokkala et al. 2015, 2017, 2019).

#### Chromosome staining techniques

##### Conventional staining

Air-dried slides were stained according to the Schiff-Giemsa protocol first developed by Puro and Nokkala (1977) and then slightly modified by Grozeva and Nokkala (1996) for the study of true bugs. In brief, slides prepared from the testes were immersed in 1N HCl at room temperature for 15 min, hydrolyzed in 1N HCl at 60 °C for 8 min and stained with Schiff’s reagent for 20 min. Unreacted Schiff’s reagent was rinsed of thoroughly with distilled water, the slides were immersed in Sorensen’s phosphate buffer, pH 6.8, for 5 min, and stained with 2% Giemsa in Sorensen’s buffer for 20–30 min. When adequate staining was achieved, the slides were rinsed briefly with distilled water, air-dried, and mounted in Entellan (a mounting medium). With slides prepared from the ovaries, a slightly modified Schiff-Giemsa method was used. Slides were subjected to hydrolysis in 1 N HCl first at room temperature for 20 min and then at 60 °C for 8 min, and stained in Schiff’s reagent for 20 min. After rinsing thoroughly in distilled water, the slides were additionally stained with 4% Giemsa in Sørensen’s buffer (pH 6.8) for 20 min. The slides were rinsed briefly in distilled water, air-dried and mounted in Entellan.

As it will be discussed in subsequent parts (papers) of the monograph, almost all Paraneoptera insects have holokinetic chromosomes that display a very limited number of distinctive characters (markers) making it difficult and often completely impossible to identify homologues in the karyotype and trace the behavior of chromosomes in meiosis and reproductive cycles in general. The search for chromosomal markers in “holokinetic insects” is therefore of particular importance. In these insects, including objects of this study, such techniques as C-banding, AgNOR-staining, DNA specific fluorochrome banding and fluorescence in situ hybridization (**FISH**) are widely used. Below, we will provide a brief description of these approaches and note their capabilities and goals in studies of karyotypes and gametogenesis in Paraneoptera.

#### Sequential staining

##### C-banding

Chromosomes are known to consist of euchromatin and heterochromatin, which have different staining properties. C-banding technique detects blocks of constitutive heterochromatin (C-bands) consisting of satellite DNAs, which are highly repetitive sequences of DNA with no known genes, and remain condensed all throughout the cell cycle. In monocentric chromosomes, C-bands are present mainly in the centromeric regions, although they may also occur at any other position along the chromosomes. In holokinetic chromosomes, C-bands are mainly confined to terminal portions of the chromosomes, although they are also present in nucleolar constrictions occupying one or both sides of the constriction (NOR-associated heterochromatin) and sometimes also in interstitial regions of the chromosome. In our studies, we used a slight variation of the conventional C-banding procedure which was developed by Sumner (1972) and up to the present time is widely used in various laboratories of the world. Slides were aged at 37 °C for 7–10 days, treated with 0.2 N HCl for 20 min at room temperature, immersed in a saturated solution of Ba(OH)_2_ at room temperature for 1 to 14 min (time depends on the object), rinsed three times in water, immersed in 2 × SSC (sodium chloride 0.3M and 0.03M trisodium citrate, pH 7.0) at 60 °C for 1 hr, thoroughly rinsed, air-dried and stained with 4–5% Giemsa solution in Sørensen’s phosphate buffer, pH 6.8. When appropriately stained, the preparations were rinsed briefly with distilled water, air dried, and mounted in Entellan.

##### AgNOR-staining

Nucleolus organizer regions (NORs), which give rise to the interphase nucleoli, are defined as nucleolar components containing ribosomal genes and the argyrophilic NOR-associated proteins (AgNOR proteins), which bind silver ions. AgNOR proteins are selectively stained by impregnation with silver nitrate (AgNO_3_) and can be identified by light microscopy as well-defined black dots exclusively localized on the NOR-carrying chromosomes and throughout the nucleolar area in interphase nuclei. The NORs stained by silver are called “AgNORs”. In our studies, we used a “one-step” silver-staining method by Howell and Black (1980), which is the most frequently employed technique for AgNOR protein visualization in routine cytogenetic studies of different eukaryotes, including insects. Slides were incubated in standard saline citrate (SSC) solution at 65 °C or in 0.2 M HCl at room temperature for 30 min and treated in 50% AgNO_3_ with gelatin as a developer (0.2 g gelatin, 10 ml distilled water, and 0.1 ml concentrated formic acid HCOOH), in the ratio 2: 1, in a moist chamber at 65 °C for 4–8 min (time is chosen empirically). The staining reaction was followed under the microscope. When the desired degree of staining was observed, the reaction was halted by rinsing with distilled water, and the preparations were dried and embedded in Entellan. The most important detail in the whole process of the experiment was to avoid the light.

### Molecular cytogenetic techniques

Fluorochrome banding and fluorescence in situ hybridization (FISH) are excellent molecular cytogenetic tools which provide various possibilities in the study of chromosome structure and genome organization and contribute to a better characterization of the karyotype and meiosis.

#### Base-specific fluorochrome staining

Constitutive heterochromatin (C-heterochromatin; see above) can be enriched with G-C (guanine-cytosine) or A-T (adenine-thymine) base pairs of DNA. The most widely used base-specific fluorochromes, CMA_3_ (chromomycin A_3_) and DAPI (4’,6-diamidino-2-phenylindole), are fluorescent dyes that bind strongly to GC-rich and AT-rich regions in DNA, respectively, and reveal, thus, the molecular composition of C-heterochromatin. Comparative patterns of fluorochrome banding allow the identification of homologous chromosomes in the karyotype. In our studies, we carried out DAPI/CMA_3_ double staining following mainly Schweizer (1980). The AT-specific fluorescent dye DAPI and GC-specific dye CMA_3_ were dissolved in Mcllvaine’s citric acid/NaHP buffer at pH 7, and in the diluted (1:1) pH 7 buffer containing 5 mM MgCl_2_, respectively. Chromosomal preparations were stained for 25–45 min with CMA_3_ (0.5 mg/ml), briefly rinsed with buffer, stained with AT-specific antibiotic distamycin A (DA) (0.1 mg/ml) for 5–15 min, again briefly washed, and finally stained with DAPI (0.6 mg/ml) for 20–30 min. To improve staining reaction, we added 5% methanol in the fluorescent staining solutions. After fluorochrome staining, slides were washed twice in 70% ethanol for 30 min and stained with 4% Giemsa for C-banding. The preparations were then rinsed with buffer, air-dried, mounted in a mixture of 70% glycerol and pH 7.0 Mcllvaine’s buffer (1:1) and sealed with rubber solution. To prevent fading of CMA_3_-fluorescence, we added 1% n-propyl-gallate in the mounting medium. Prior to examination, the preparations were stored in the dark for several days, by which time both the chromomycin A_3_ and DAPI fluorescence are stabilized.

#### Fluorescence in situ hybridization (FISH)

FISH technique, developed about 50 years ago (Gall and Pardue 1969; John et al. 1969), is powerful for the physical mapping of genes and defined DNA sequences directly on chromosomes by hybridization of complementary fluorescently labeled DNA probes on cytological preparations. This technique is very helpful in chromosome-based genome assemblies, providing information on the fine architecture of genomes and their evolution. In our studies, we mainly used two-color FISH for mapping the multigene family of rDNA and the insect-type telomeric motif (TTAGG)*_n_*. We aimed to study the number and the distribution of rDNA loci and use them as markers for the identification of specific chromosomes and comparative chromosome mapping as well as for tracing chromosome behavior during meiosis and gametogenesis in general. Another goal was to find out whether a particular taxon has retained the evolutionarily ancestral “insect” motive of telomeres (TTAGG)*_n_*, and, if not, at what stages of the evolution losses, gains or changes of this motif happened. We have developed and published detailed FISH protocols (Grozeva et al. 2015; Kuznetsova et al. 2015) specific to several model hemipteroid species, including the common bedbug *Cimex lectularius* Linnaeus, 1758 (Heteroptera, Cimicidae) and the representatives of the spittlebug genus *Philaenus* Stål, 1864 (Auchenorrhyncha, Aphrophoridae), which is taxonomically challenging due to outstanding color polymorphism of the species involved (e.g. Drosopoulos et al. 2010). Although these protocols were developed for *C. lectularius* and *Philaenus* spp., they have been successfully used since then for many other hemipteran insects (see e.g., Golub et al. 2018, 2019; Maryańska-Nadachowska et al. 2018).

The target chromosome preparations were prepared some time prior to hybridization to allow thorough drying and aging of the chromatin on the slide by incubation at 60 °C for at least a few hours. The 18S rDNA probe was amplified by PCR and labelled with biotin-11-dUTP (Fermentas, Vilnius, Lithuania) using genomic DNA of the true bug *Pyrrhocoris apterus* (Linnaeus, 1758): an initial denaturation period of 3 min at 94 °C was followed by 33 cycles of 30 s at 94 °C, annealing for 30 s at 50 °C and extension for 1.5 min at 72 °C, with a final extension step of 3 min at 72 °C. The telomere probe (TTAGG)_n_ was amplified by PCR and labeled with rhodamine-5-dUTP (GeneCraft, Köln, Germany): an initial denaturation period of 3 min at 94 °C was followed by 30 cycles of 45 s at 94 °C, annealing for 30 sec at 50 °C and extension for 50 sec at 72 °C, with a final extension step of 3 min at 72 °C. The chromosome preparations were treated with 100 μg/ml RNase A and 5 mg/ml pepsin solution to remove excess RNA and proteins. Chromosomes were denatured in the hybridization mixture containing labeled 18S rDNA and (TTAGG)*_n_* probes with an addition of salmon sperm DNA blocking reagent and then hybridized for 42 h at 37 °C. 18S rDNA probes were detected with NeutrAvidin-Fluorescein conjugate (Invitrogen, Carlsbad, CA, USA). The chromosomes were mounted in an antifade medium (ProLong Gold antifade reagent with DAPI, Invitrogen) and covered with a glass coverslip.

## Terminology

Considering that the terminology used to describe different aspects of reproductive biology and ontogenesis is not very well known to entomologists, and in the same time the meaning of individual terms varies in the literature, below we provide an annotated list of the most important terms used in this field.

**Arrhenotoky** – parthenogenetic mode where females produce only males from unfertilized eggs). There are two forms: haplodiploidy (males are haploid due to direct development from haploid eggs) and diploid arrhenotoky (males develop from diploid eggs, similar to automictic thelytoky).

**Contagious parthenogenesis** – a process involving rare functional males produced by a parthenogenetic lineage, which mate with bisexually reproducing females resulting in fertile parthenogenetic offspring.

**Cyclic parthenogenesis** – the regular alternation of bisexual and parthenogenetic reproduction in the same species.

**Deuterotoky** – parthenogenetic mode where females and males are produced from unfertilized eggs.

**Exuviatrium** – sclerotized larval exuvium, which is used by the next larva-like instar (including neotenic female) as a shelter. ***Exuviatrial*** female has minute, rudimentary legs and lays eggs just inside exuviatrium. The term was introduced by Gavrilov-Zimin (2018).

**Gynandromorphism** – the phenomenon by which an individual is a sexual mosaic exhibiting characters of both sexes in various parts of the body; ***bilateral gynandromorphs*** are insects with male and female tissues distributed nearly bilaterally.

**Larva** – preadult instar of postembryonal development. Different instars are usually designated by numbers (I, II, III, IV, etc.) according to the number of molts which the animal underwent after the birth. Kluge (2010b) suggested using special Latin names for such instars: ***primolarva***, ***secundolarva***, ***tertiolarva***, etc. In the situations when the total number of the instars is unknown he recommended naming instars, starting from the oldest one: ***ultimolarva*** (preadult instar), ***penultimolarva***, etc.

**Neoteny** – bisexual reproduction of preimaginal instars. The term was originally introduced by Kollmann (1884) for salamanders, but now is widely used for different vertebrate and invertebrate animals.

**Nymph** – larval instar with wing buds (*protoptera*). These instars can also be named as ***primo***-, ***secundo***-, ***tertio***-, ***ultimo***-, ***penultimo***-nymphs, etc.

**Occasional eggs retention** – occasional cases of starting the embryonic development inside mother’s body due to unpredictable reasons, such as premature death of the mother, sudden change of environmental conditions, etc.

**Oviparity** – laying eggs before starting of embryogenesis; all embryonal development occurs outside the mother’s body.

**Ovoviviparity** – laying eggs with fully or partly developed embryo inside; embryo starts to develop inside the mother’s body; egg is covered with a chorion and contains sufficient yolk to nourish the embryo until hatching without receiving aliment from the maternal organism.

**Complete ovoviviparity** – laying eggs with fully developed embryo inside; hatching of the primolarva occurs just after the oviposition.

**Incomplete ovoviviparity** – laying eggs with partly developed embryo inside; hatching of the primolarva occurs sometimes after oviposition.

**Facultative ovoviviparity** – individual and geographical variation at the stage of the embryonal development inside of laying egg, from cleavage divisions to complete embryogenesis.

**Obligate ovoviviparity** – invariable laying egg at a certain stage of embryonal development in all specimens of a taxon.

**Paedogenesis** – parthenogenetic reproduction of preimaginal instars. The term was introduced by Baer (1866) for larval parthenogenesis of some Cecidomyiidae (Diptera) discovered by Wagner (1862).

**Paternal genome elimination** (PGE) – a mode of reproduction where only the female genome is transmitted to offspring (sometimes also referred to as pseudo-arrhenotoky or parahaploidy). Paternal genome set is eliminated or inactivated in early embryogenesis (males are somatically haploid) or during spermatogenesis (males are somatically diploid; however, the paternal genome is eliminated, partly or totally inactivated by chromatin condensation, also referred to as paternal genome heterochromatinization).

**Ploidy restoration** – a process accompanying meiosis during automictic parthenogenetic development. There are three mechanisms known to date: ***premeiotic doubling*** of chromosomes with standard meiosis afterwards; ***postmeiotic restoration*** where haploid ootids fuse and produce a diploid nucleus (also known as ‘central fusion’); and ***meiotic restoration*** – fusion of secondary oocytes with second polar body following second meiotic division.

**Protopteron** (plural **protoptera**) – wing buds, flattened structures possessed by nymphs from which the wings will develop into imago. The term was introduced by Kluge (2005, 2010a, b).

**Pseudopuparium** – immovable apodal ultimolarva of whiteflies (Homoptera: Aleyrodinea); this pseudopuparium does not have protoptera (wing buds), but molts to a winged imago having well-developed legs and antennae. In contrast to the true puparium (Cyclorrhapha and Strepsiptera; Diptera), there is no pupa inside ultimolarval exuvium of whiteflies, and imaginal cuticle is forming just under larval cuticle.

**Puparium** – larval exuvium which covers a pupa, quiescent instar which molts to imago.

**Thelytoky** – parthenogenetic mode where females produce only females from unfertilized eggs. There are two forms described in animals – apomixis and automixis.

- **apomixis** – a mode where a single mitotic-like division in unfertilized eggs results in genetic identity of the mother and her offspring (=ameiotic parthenogenesis).

- **automixis** – a mode where egg cells are produced by meiosis, the diploid state of the offspring being restored by the fusion of meiotic products.

**Viviparity** – laying primolarvae, which are not covered by a chorion (Fig. 1d); whole embryogenesis occurs inside the mother’s body with receiving nutriment via special maternal placenta-like structures or from other organs of embryonal and/or maternal origin. Of the different distinguishable variants of true viviparity (Hagan 1951), Paraneoptera were suggested to have the so-called (***pseudo***)***placental viviparity*** in viviparous species. In this case, embryonic and/or maternal tissues form a placenta-like structure for embryo nourishment (see the second chapter of this monograph). It seems however that there are no fundamental differences between terms “placenta” and “pseudoplacenta”; these morphologically similar structures have arisen many times independently in different phylogenetic lines of invertebrates and viviparous vertebrates.

## Higher classification and nomenclature

The system of higher taxa names, used in this monograph, follows hierarchical rank and typified (for superfamily and lower rank names) nomenclature. For taxa of rank above the suborder, circumscriptional names are used, based on their priority. For taxa of the suborder and infraorder ranks, circumscriptional names are used inside Copeognatha, Parasita, and Thysanoptera, whereas the typified names are used inside Arthroidignatha (=Hemiptera sensu stricto). The system and comments are adopted mainly from Gavrilov-Zimin and Danzig (2012) and Gavrilov-Zimin (2018); however, some conflicting approaches are also mentioned.

The widely known name Paraneoptera Martynov, 1923 was originally introduced with an uncertain inclusion of Zoraptera Silvestri, 1913, but subsequently Martynov (1938) explicitly placed this order in Paraneoptera.

The name Copeognatha Enderlein, 1903 is only one year older than the name Psocoptera Shipley, 1904, used for the same taxon. Another widely used name Corrodentia Burmeister, 1839 was proposed originally for a polyphyletic taxon including not only psocids, but also unrelated Polyneoptera and Neuroptera insects (Isoptera + Embioptera and Conyopterigidae, respectively).

The oldest name covered all lice is Parasita Latreille, 1796, which has priority over the frequently used names Anoplura Leach, 1815 and Phthiraptera Haeckel, 1896. Moreover, the last name was originally proposed for sucking lice only being, thus, a junior synonym of Siphunculata Latreille, 1802 (see for details Kluge 2020: 536, 545).

The name Hemiptera Linnaeus, 1758, frequently used in the literature as an order name for all “rhynchotous” insects, is nowadays a very ambiguous term since: 1) this name was used by C. Linnaeus for “rhynchotous” + thrips together; therefore it is an older synonym for Condylognatha Börner, 1904; 2) for many years up to now, this name has been used by numerous authors for true bugs (Heteroptera) only; 3) there are at least two separate orders (Heteroptera and Homoptera) within the “order Hemiptera” accepted by different authors. A similar taxonomic ambiguity concerns the well-known and widely used name Rhynchota Burmeister, 1835, which originally also included Siphunculata. Moreover, this name is preoccupied by Rhynchota Billberg, 1820 (=Aphaniptera Kirby et Spence, 1815) (Kluge 2010a). The oldest name for the taxon [aphids + scale insects + whiteflies + psyllids + cicadas + true bugs + moss bugs] is Arthroidignatha Spinola, 1850 (Kluge 2000, 2010a, b, 2020).

As for the widely known and frequently discussed order name Homoptera Latreille, 1810, there is no good reason to reject it. It originally covered all hemipteroid insects without true bugs but with thrips. However, all subsequent authors accepted this group without thrips, and Westwood (1838) seems to be the first who did it. Later, Pearce (1936) introduced the name Homopterida for the same group of taxa (i.e. without thrips). The concept of Homoptera sensu Westwood, 1838 as a paraphyletic group (for review, see for example, Dohlen and Moran 1995 or Gullan 1999), takes into account some facts and ignores others. According to cladistics, the problem comes down to considering synapomorphies of the Hemelytrata Fallén, 1829 (Cicadinea + Coleorrhyncha + Heteroptera) in contrast to synapomorphies of the Homoptera. Some authors (e.g., Gullan 1999) suppose that Homoptera are characterized by plesiomorphic characters only. Indeed, it is not easy to find reliable synapomorphies for all very diverse groups of Homoptera. However, such characters as the wing-coupling apparatus, the presence of the fields of wax glands and filter chamber of the digestive tract as well as the ability to produce honeydew can be considered as synapomorphies of Homoptera (Lambdin 2001; D’Urso 2002; Gavrilov-Zimin and Danzig 2012; Gavrilov-Zimin 2020). There is no reason to ignore these characters and consider only the probable morphological synapomorphies of Hemelytrata (see, e.g., Emeljanov 1987; Kluge 2020) or accept unconditionally untestable and controversial molecular cladograms based on a small number of occasionally selected species. Some of these cladograms (Campbell et al. 1995; Dohlen and Moran 1995; Johnson et al. 2018) support Homoptera as a paraphyletic group, whereas others (e.g. Song et al. 2012) – as a holophyletic one. A detailed historical revision of different phylogenetic reconstructions of “rhynchotous” insects was given by Brożek et al. (2003) and Forero (2008) and therefore will not be repeated here. In any case, regardless of whether further investigations will support or not the paraphyly of the Homoptera, there is no reason to reject this taxonomic name. Cladistic rejecting paraphyletic taxa is based not on scientific arguments but on voluntarism. There is no biological reason to suppose that species in paraphyletic taxa should be less related to each other than those in holophyletic taxa. This main conceptual contradiction between cladistic taxonomy (in its original W. Hennig’s sense) and evolutionary taxonomy has been addressed in many publications (e.g., Simpson 1961; Mayr 1974; Mayr and Ashlock 1991; Gorochov 2001; Kerzhner and Danzig 2001; Hołyński 2005; Rasnitsyn 2010). Moreover, paraphyly of a taxon is closely connected with our subjective view of taxon boundaries. For example, if we include fossil ancestor groups of Arthroidignatha (in particular, Archescytinoidea) in Homoptera, the latter will evidently be paraphyletic; on the other hand, if we include Archescytinoidea in Hemelytrata (Cicadinea+Coleorrhyncha+Heteroptera), the latter will be paraphyletic. The factual paleontological data on the appearance of different Arthroidignatha groups are provided in the scheme of Shcherbakov and Popov (2002).

Concerning the frequently used name Sterno(r)rhynch(i)(a) (= Coccinea + Aphidinea + Aleyrodinea + Psillinea), we are not sure about the commonly discussed synapomorphies of this group. For example, according to the Shcherbakov and Popov’ scheme (2002), Sternorhynchi are polyphyletic. Moreover, Sternorhynchi Amyot et Serville, 1843 is a junior synonym of Plantisuga Dumeril, 1805 (Kluge 2010a).

Summarizing all of the above, we recognize scale insects, aphids, psyllids, whiteflies and cicadas as suborders of the order Homoptera sensu Westwood, 1838, and use the ending “-nea” for all typified suborder names within Homoptera (Aphidinea, Coccinea, Aleyrodinea, Psyllinea, Cicadinea) following Pesson (1951). The International Code of Zoological Nomenclature does not regulate the taxonomic names above “family group” and we follow the principle introduced by Rohdendorf (1977) and consider the suborder names as the family-group ones.

**Supercohors Paraneoptera Martynov, 1923**

Ordo **Zoraptera** Silvestri, 1913

Cohors **Acercaria** Börner, 1904

Superordo **Panpsocoptera** Crampton, 1938

Ordo **Copeognatha** Enderlein, 1903 (= Psocoptera Shipley, 1904)

Ordo **Parasita** Latreille, 1796

Subcohors **Hemiptera** Linnaeus, 1758 (= Condylognatha Börner, 1904, non Hemiptera auct.)

Ordo **Thysanoptera** Haliday, 1836

Superordo **Arthroidignatha** Spinola, 1850 (= Hemiptera auct., non Linnaeus, 1758;

= Rhynchota auct., non Burmeister, 1835)

Ordo **Coleorrhyncha** Meyers et China, 1929

Ordo **Heteroptera Latreille, 1810** (= Hemiptera auct., non Linnaeus, 1758)

Ordo **Homoptera** sensu Westwood, 1838, non Latreille, 1810 (= Homopterida Pearce, 1936)

Subordo **Cicadinea** Batsch, 1789

Subordo **Psyllinea** Latreille, 1807

Subordo **Aleyrodinea** Newman, 1834

Subordo **Aphidinea** Latreille, 1802

Superfamilia **Phylloxeroidea** Herrich-Schaeffer, 1854

Superfamilia **Aphidoidea** Latreille, 1802

Subordo **Coccinea Fallén, 1814** (= Coccoidea auct., Gallinsecta De Geer, 1776)

Superfamilia **Orthezioidea Amyot et Serville, 1843** (=Paleococcoidea Borchsenius, 1950; = Archeococcidea Bodenheimer, 1952)

Superfamilia **Coccoidea Fallén, 1814** (=Neococcoidea Borchsenius, 1950; = Neococcidea Bodenheimer, 1952)

## Author contributions

I.G.-Z. and V.K. contributed equally to the paper; they conceived and designed the project and prepared the manuscript draft. I.G.-Z. also wrote the section “Higher classification and nomenclature”. S.G. edited and commented on the draft manuscript and also prepared some paragraphs in the section Material & Methods. D.G. and A.K. collected and identified part of the material, provided some illustrations and made several additions to the text. K.T. collected and identified part of the material.

## References

[B1] Baer KM (1866) About Professor N. Wagner’s discovery of agamic reproduction of larvae, about additional observations of this phenomenon by Mr. Ganin, and about paedogenesis in general. Notes of Academy of Sciences (St. Petersburg) 10(Supplement 1): 1–77. [In Russian] [Version in German: Baer KH (1866) Ueber Prof. Nic. Wagners’s Entdeckung von Larvae die sich fortpflanzen. Herr Ganin’s verwandte und ergänzende Beobachtungen und über dei Paedogenese überhaupt. Bulletin de l’Académie Impériale des Sciences de St. Petersburg 9: 64–137]

[B2] Brożek J Szwedo J Gaj D Pilarczyk S (2003) Former and current views on theclassification of the bugs (Insecta, Hemiptera). Genus. Supplement I: 85–100.

[B3] Campbell BC Steffen–Campbell JD Sorensen JT Gill RJ (1995) Paraphyly of Homoptera and Auchenorrrhyncha inferred from 18 S rDNA nucleotide sequences. Systematic Entomology 20: 175–194. 10.1111/j.1365-3113.1995.tb00090.x

[B4] Conger AD Fairchilld M (1953) A quick-freeze method for making smear slides permanent. Stain Technology 28: 289–293. 10.3109/10520295309105557 13113454

[B5] De Meeûs T Prugnolle F Agnew P (2007) Asexual reproduction: genetics and evolutionary aspects. Cellular and Molecular Life Sciences 64: 1355–1372. 10.1007/s00018-007-6515-2 17396223 PMC11136054

[B6] Dikshith TSS (1964) Chromosome behaviour in *Laccifer lacca* (Kerr) Lacciferidae–Coccoidea. Cytologia 29: 337–345. 10.1508/cytologia.29.337

[B7] Dohlen CD von Moran NA (1995) Molecular phylogeny of the Homoptera: a paraphyletic taxon. Journal of Molecular Evolution 41: 211–223. 10.1007/BF00170675 7666451

[B8] Drosopoulos S Maryańska-Nadachowska A Kuznetsova VG (2010) The Mediterranean: area of origin of polymorphism and speciation in the spittlebug *Philaenus* (Hemiptera, Aphrophoridae). Zoosystematics and Evolution 86(1): 125–128. 10.1002/zoos.200900017

[B9] D’Urso V (2002) The wing–coupling apparatus of HemipteraAuchenorrhyncha: structure, function, and systematic value. Denisia 4: 401–410.

[B10] Emeljanov AF (1987) Phylogeny of cicads (Homoptera, Cicadina) based on comparative morphological studies. Trudy Vsesoyuznogo Entomologicheskogo Obschestva 69: 19–109. [In Russian]

[B11] Essig EO (1948) Mounting aphids and other small insects on microscopic slides. Pan–Pacific Entomologist 24: 9–22.

[B12] Forero D (2008) The systematics of the Hemiptera. Revista Colombiana de Entomologia 34(1): 1–21.

[B13] Gall JG Pardue ML (1969) Formation and detection of RNA-DNA hybrid molecules in cytological preparations. Proceedings of the National Academy of Sciences of the United States of America 63(2): 378–383. 10.1073/pnas.63.2.378 4895535 PMC223575

[B14] Gapon DA (2001) Inflation of heteropteran aedeagi using microcapillaries (Heteroptera, Pentatomidae). Zoosystematica Rossica 9(1): 157–160. [2000]

[B15] Gavrilov-Zimin IA (2018) Ontogenesis, morphology and higher classification of archaecococcids (Homoptera: Coccinea: Orthezioidea). Zoosystematica Rossica (Supplementum 2), 260 pp. 10.31610/zsr/2018.supl.2.1

[B16] Gavrilov-Zimin IA (2020) Homologous series by Nikolai Vavilov in the phylogeny of Homoptera. Comparative Cytogenetics 14(4): 589–596. 10.3897/CompCytogen.v14.i4.60894 33376584 PMC7759555

[B17] Gavrilov-Zimin IA Danzig EM (2012) Taxonomic position of the genus *Puto* Signoret (Homoptera: Coccinea: Pseudococcidae) and separation of higher taxa in Coccinea. Zoosystematica Rossica 22(1): 97–111. 10.31610/zsr/2012.21.1.97

[B18] Gokhman VE Kuznetsova VG (2018) Parthenogenesis in Hexapoda: holometabolous insects. Journal of Zoological Systematics and Evolutionary Research 56: 23–34. 10.1111/jzs.12183

[B19] Golub N Anokhin B Kuznetsova V (2019) Comparative FISH mapping of ribosomal DNA clusters and TTAGG telomeric sequences to holokinetic chromosomes of eight species of the insect order Psocoptera. Comparative Cytogenetics 13(4): 403–410. 10.3897/CompCytogen.v13i4.48891 31850138 PMC6910881

[B20] Golub NV Golub VB Kuznetsova VG (2018) New data on karyotypes of lace bugs (Tingidae, Cimicomorpha, Hemiptera) with analysis of the 18S rDNA clusters distribution. Comparative Cytogenetics 12(4): 515–528. 10.3897/CompCytogen.v12i4.30431 30588289 PMC6302064

[B21] Gorochov AV (2001) On some theoretical aspects of taxonomy (remarks by the practical taxonomist). Acta Geologica Leopoldensia 24(52/53): 57–71.

[B22] Grozeva S Nokkala S (1996) Chromosomes and their meiotic behaviour in two families of the primitive infraorder Dipsocoromorpha (Heteroptera). Hereditas 125: 31–36. 10.1111/j.1601-5223.1996.t01-1-00031.x

[B23] Grozeva S Anokhin B Kuznetsova VG (2015) Bed bugs (Hemiptera), Chapter 8. In: Sharachov I (Ed.) Protocols for Cytogenetic Mapping of Arthropod Genomes.CRC press, Taylor & Francis Group, Boca Raton, 285–326. 10.1201/b17450-9

[B24] Gullan PJ (1999 [2001] ) Why the taxon Homoptera does not exist. Entomologica (Bari) 33: 101–104.

[B25] Hagan HR (1951) Embryology of viviparous insects. New York, 472 pp.

[B26] Henry ThJ (2017) Biodiversity of Heteroptera. In: Robert GF, Peter HA (Eds) Insect Biodiversity: Science and Society I, 279–335. 10.1002/9781118945568.ch10

[B27] Hołyński RB (2005) Philosophy of science from taxonomist’s perspective. Genus 16(4): 469–502.

[B28] Howell WM Black DA (1980) Controlled silver staining of nucleolus organizer regions with protective colloidal developer: a 1-step method. Experientia 36: 1014–1015. 10.1007/BF01953855 6160049

[B29] Ivanova-Kazas OM (1995) Evolutional embryology of animals. St. Petersburg, 565 pp. [In Russian]

[B30] John HA Birnstile ML John KW (1969) RNA-DNA hybrids at the cytological level. Nature 223: 582–587. 10.1038/223582a0 5799530

[B31] Johnson KP Dietrich CH Friedrich F Beutel RG Wipfler B Peters RS Allen JM Petersen M Donath A Walden KKO Kozlov AM Podsiadlowski L Mayer C Meusemann K Vasilikopoulos A Waterhouse RM Cameron SL Weirauch C Swanson DR Percy DM Hardy NB Terryr I Lius S Zhout X Misof B Robertson HM Yoshizawa K (2018) Phylogenomics and the evolution of the hemipteroid insects. PNAS 115: 12775–12780. 10.1073/pnas.1815820115 30478043 PMC6294958

[B32] Kerzhner I Danzig E (2001[2002]) Hemiptera, Homoptera, Sternorrhyncha. Bollettino di Zoologia Agraria e di Bachicoltura (Milano) 33(3): 217–218.

[B33] Kluge NJ (2000) Modern systematics of insects. I. St. Petersburg, 336 pp. [In Russian]

[B34] Kluge NJ (2005) Larval/pupal leg transformation and a new diagnosis for the taxon Metabola Burmeister, 1832 = Oligoneoptera Martynov, 1923. Russian Entomological Journal 13(4): 189–229.

[B35] Kluge NJ (2010a) Circumscriptional names of higher taxa in Hexapoda. Bionomina, 1: 15–55. 10.11646/bionomina.1.1.3

[B36] Kluge NJ (2010b) Paradoxical molting process in *Orthezia urticae* and other coccids (Arthroidignatha, Gallinsecta). Zoosystematica Rossica 19(2): 246–271. 10.31610/zsr/2010.19.2.246

[B37] Kluge NJ (2020) Insect systematics and principles of cladoendesis. In 2 volumes. KMK Scientific Press, Moscow, 1037 pp. [In Russian]

[B38] Kollmann J (1884[1885]) Das Überwintern von europäischen Frosch – und Tritonlarven und die Umwandlung des mexicanischen Axolotle. Verhandlungen Naturforschende Gesellschaft (Basel) 7: 387–398.

[B39] Kuznetsova VG Maryańska-Nadachowska A Karamysheva TV (2015) Spittlebugs, Chapter 10. In: Sharakhov IV (Ed.) Protocols for Cytogenetic Mapping of Arthropod Genomes.CRC press, Taylor & Francis Group, Boca Raton, 351–380. 10.1201/b17450-9

[B40] Lambdin PL (2001[2002] ) Discourse on the classification of the scale insects. Bollettino di Zoologia Agraria e di Bachicoltura (Milano) 33(3): 209–213.

[B41] Leather SR Hardie J (2017) Insect reproduction, Boca Raton, 266 pp. 10.1201/9781351073608

[B42] Martynov AV (1938) Etudes sur l’histoire geologique et de phylogenie des ordres des Insectes (Pterygota) I. Palaeoptera et Neoptera-Polyneoptera. Travaux de l’Institute Paléontologique de l’Academie des Sciences de l’URSS 7(4): 1–449. [In Russian]

[B43] Maryańska-Nadachowska A Kuznetsova VG Golub NV Anokhin BA (2018) Detection of telomeric sequences and ribosomal RNA genes in holokinetic chromosomes of five jumping plant-lice species: First data on the superfamily Psylloidea (Hemiptera: Sternorrhyncha). European Journal of Entomology 115: 632–640. 10.14411/eje.2018.061

[B44] Mayr E (1974) Cladistic analysis of cladistic classification? Zeitschift Zoologische Systematik und Evolution 12(2): 94–128. 10.1111/j.1439-0469.1974.tb00160.x

[B45] Mayr E Ashlock NY (1991) Principles of systematic zoology. NewYork, 475 pp.

[B46] Nokkala C Kuznetsova VG Nokkala S (2015) Rare diploid females coexist with rare males: a novel finding in triploid parthenogenetic populations in the psyllid Cacopsylla myrtilli (W. Wagner, 1947) (Hemiptera, Psylloidea) in northern Europe. Genetica 143: 589–595. 10.1007/s10709-015-9858-x 26208490

[B47] Nokkala S Kuznetsova V Nokkala Ch (2017) Characteristics of parthenogenesis in *Cacopsylla ledi* (Flor, 1861) (Hemiptera, Sternorryncha, Psylloidea): cytological and molecular approaches. Comparative Cytogenetics 11(4): 807–817. 10.3897/compcytogen.v11i4.21362 29302299 PMC5740394

[B48] Nokkala Ch Kuznetsova V Rinne V Nokkala S (2019) Description of two new species of the genus *Cacopsylla* Ossiannilsson, 1970 (Hemiptera, Psylloidea) from northern Fennoscandia recognized by morphology, cytogenetic characters and COI barcode sequence. Comparative Cytogenetics 13: 367–382. 10.3897/CompCytogen.v13i4.47395 31798796 PMC6879664

[B49] Pearse AS (1936) Zoological names. A list of phyla, classes, and orders. Durham, 24 pp.

[B50] Pesson P (1951) Ordre des Homoptères and Ordre des Thysanopteres. In: Grassé PP (Ed.) Traité de Zoologie: Anatomie, Systématique, Biologie.[Vol. X. Insectes Supérieurs et Hémiptéroïdes (Fasc. 11).], Paris, 1341–1364, 1805–1869.

[B51] Poisson R Pesson P (1951) Super-Ordre des Hémiptéroides. Généralités. In: Grassé PP (Ed.) Traité de Zoologie: Anatomie, Systématique, Biologie.[Vol. X. Insectes Supérieurs et Hémiptéroïdes (Fasc. 11).], Paris, 1385–1389.

[B52] Puro J Nokkala S (1977) Meiotic segregation of chromosomes in *Drosophila melanogaster* oocytes. Chromosoma 63: 273–286. 10.1007/BF00327454

[B53] Rasnitsyn AP (2010) Molecular phylogenetics, morphological cladistics and fossils. Entomological Review 89(1): 85–132. 10.1134/S0013873810030012

[B54] Rohdendorf BB (1977) The rationalization of names of higher taxa in zoology. Paleontological Journal 11: 149–155.

[B55] Romeis B (1953) Microscopic technics. Moscow, 718 pp. [In Russian]

[B56] Shcherbakov DE Popov YuA (2002) Superorder Cimicidea. In: Rasnitsyn AP Quicke DLJ (Eds) History of insects.New York, Boston, Dordrecht, London, Moscow, 143–157.

[B57] Schweizer D (1980) Simultaneous fluorescent staining of R bands and specific heterochromatic regions (DA-DAPI bands) in human chromosomes. Cytogenetics and Cell Genetics 27(2–3): 190–193. 10.1159/000131482 6156801

[B58] Simon JC Delmotte F Rispe C Crease T (2003) Phylogenetic relationships between parthenogens and their sexual relatives: the possible routes to parthenogenesis in animals. Biological Journal of the Linnean Society 79: 151–163. 10.1046/j.1095-8312.2003.00175.x

[B59] Simpson GG (1961) Principles of animal taxonomy. New York, 247 pp. 10.7312/simp92414

[B60] Song N Liang A-P Bu C-P (2012) A Molecular phylogeny of Hemiptera inferred from mitochondrial genome sequences. PLoS ONE 7(11): e48778. 10.1371/journal.pone.0048778 PMC3493603 23144967

[B61] Sumner AT (1972) A simple technique for demonstrating centromeric heterochromatin. Experimental Cell Research 75(1): 304–306. 10.1016/0014-4827(72)90558-7 4117921

[B62] Vershinina AO Kuznetsova VG (2016) Parthenogenesis in Hexapoda: Entognatha and non-holometabolous insects. Journal of Zoological Systematics and Evolutionary Research 54: 257–268. 10.1111/jzs.12141

[B63] Wagner NP (1862) Spontaneous reproduction in insect larvae. Kazan’, 50 pp. [In Russian]

[B64] Westwood JO (1838) The entomologist’s text book; an introduction to the natural history, structure, physiology, and classification of insects. London, 432 pp. 10.5962/bhl.title.39827

[B65] White MJD (1973) Animal Cytology and Evolution. Cambridge, 961 pp.

